# Changes in the spectrum of kidney diseases: a survey of 2803 patients from 2010 to 2018 at a single center in southeastern China

**DOI:** 10.1080/0886022X.2022.2083517

**Published:** 2022-06-03

**Authors:** Linfeng Zheng, Luxia Tu, Haowen Huang, Li Zhang, Ying Wang, Jing Zhou, Qinkai Chen, Xin Wei

**Affiliations:** a Department of Nephrology, The First Affiliated Hospital of Nan Chang University, Nanchang, China; b Department of Pathology, The First Affiliated Hospital of Nanchang University, Nanchang City, China; c Department of Nephrology, People’s Hospital of Ganjiang New District, Nanchang, China

**Keywords:** Primary glomerulonephritis, secondary glomerulonephritis, idiopathic membranous nephropathy, IgA nephropathy, renal biopsy

## Abstract

Primary glomerular disease was the leading cause of chronic kidney disease (CKD) in China; however, changes in the economy and environment introduce variations in the spectrum of kidney diseases. This study aimed to analyze renal biopsy data to inform disease prevention and public health interventions. In this retrospective cohort study, data from 2,803 consecutive renal biopsies conducted at our center between January 2010 and December 2018 were analyzed. The sample was disaggregated by age and the date of biopsy to facilitate analysis. Primary glomerulonephritis (PGN) is the most frequent (81.84%) finding, followed by secondary glomerulonephritis (SGN; 15.38%), tubulointerstitial nephritis (15.38%), and others (1.57%). IgA nephropathy (IgAN), idiopathic membranous nephropathy (iMN), and minimal change disease were the primary causes of PGN. Among PGN cases, the incidence of iMN arose, especially among those aged ≥ 60 years old, during the observation period. Contrary to the case of iMN, the proportion of IgAN in PGN trended downward, continuously, and at length. Moreover, IgAN mainly affected those aged 25–44 years old and less so those aged ≥ 60 years old. Lupus nephritis, Henoch–Schönlein purpura nephritis, and diabetic nephropathy (DN) were key causes of SGN. A ratio reversal between infectious disease and chronic disease dramatically changed SGN patterns. In the past year, the incidence of hepatitis B–related nephritis has constantly declined; however, the proportion of DN among SGN had steadily increased. The incidence of iMN significantly increased during these years. Among SGN cases, the proportion of DN has increased.

## 1. Introduction

Over the past few decades, the prevalence of chronic kidney disease (CKD) has increased sharply, at approximately 10%, in both developed and developing countries [1,2]. Because of its high prevalence and associated high medical costs, CKD has been recognized as a major global health problem. The prevention of CKD can have a remarkable effect on reducing the burden on the governments and improving public health. Therefore, effective prevention and management of the etiologies of CKD are essential. In developed countries, the leading causes of CKD are diabetes and hypertension, which is different from that in developing countries [3]. Previous studies have indicated that the major etiologies of CKD in China were primary glomerulonephritis (PGN), such as IgA nephropathy (IgAN), non-IgA mesangial proliferative glomerulonephritis (MsPGN), and minimal change disease (MCD) [4].

However, with the increasing incidence of hypertension, obesity, and diabetes mellitus; an aging population; and environmental pollution, the spectrum of CKD has changed significantly over the past several decades [1,5]. To date, although PGN continues to be the most common cause of CKD, secondary reasons are also of significance [4]. Zhou FD et al. reported that from 1993 to 2007, the spectrum of PGN changed dramatically, as the frequency of non-IgA MsPGN, endocapillary proliferative glomerulonephritis (EnPGN), and membranoproliferative glomerulonephritis (MPGN) decreased significantly, while that of MCD and IgAN increased significantly within the past 15 years [6]. In addition, a subsequent study conducted at the same center from 2003 to 2012 revealed that the frequency of idiopathic membranous nephropathy (iMN) doubled from 16.8 to 29.35% [7]. Moreover, a study from the southeastern USA reported that the frequency of diabetic glomerulosclerosis increased dramatically over the past three decades (1986–2015), suggesting that the frequency of other common glomerular diseases remained stable or has declined [8]. The temporal trends varied in different areas and races because of genetic variants, socioeconomic status, and living environments [9,10].

This retrospective study was conducted at the Department of Nephrology at the First Affiliated Hospital of Nanchang University, which is one of the largest nephropathology centers in southeastern China; the center has provided nephropathology services to patients who live throughout the Jiangxi province since the 1990s. In this study, we analyzed data of renal biopsies that were performed at our center from January 2010 to December 2018 to explore the changing spectrum of kidney disease in Jiangxi province.

## 2. Materials and methods

A total of 2906 cases were reviewed at the Department of Nephrology, the first affiliated hospital of Nanchang University, between January 1, 2010, and December 31, 2018. Clinical records were reviewed to collect clinical and laboratory information, including demographic information (age and sex) and other nephritic pathological diagnoses. These cases were rechecked by a group of technicians responsible for determining patient eligibility; patients aged <14 years were excluded, as were those with missing demographic data, renal transplant recipients, and biopsies containing <10 glomeruli under the microscope.

Standard light, immunofluorescence, and electron microscopy were routinely performed by experienced nephrologists to pathologically evaluate biopsy specimens. Pathological classification was based on the 1995 World Health Organization’s revised criteria for the classification of glomerular disease pathology. In this study, PGN was classified into IgAN, iMN, MCD, EnPGN, and others, which included MPGN, focal segmental glomerulosclerosis, and non-IgA MsPGN. Additionally, secondary glomerulonephritis (SGN) was classified into lupus nephritis (LN), Henoch–Schönlein purpura nephritis (HSPN), diabetic nephropathy (DN), hepatitis B–related nephritis (HBVN), and others, which included renal amyloidosis, hypertensive nephropathy, vasculitic renal damage, and monoclonal immunoglobulin deposition disease. Tubulointerstitial nephritis (TIN) included acute tubular necrosis, Sjögren syndrome injury, epidemic hemorrhagic fever injury, acute interstitial nephritis and pyelonephritis. If a patient had a diverse biopsy diagnosis, they were classified into their respective category by duplicate counting.

Data were categorized, based on when the biopsy was performed, to consecutive 3-year time intervals, including 2010–2012 (period 1), 2013–2015 (period 2), and 2016–2018 (period 3) for tabular presentation and data analysis. The four groups were divided according to the age when patients received renal biopsy: 14–24 years, 25–44 years, 45–59 years, ≥60 years.

This study conforms to the tenets of the Declaration of Helsinki (as revised in Brazil in 2013) of the World Medical Association. The study was approved by the Medical Ethical Committee of the first affiliated hospital of Nanchang University (approval ID [2019] 088). All patients had signed the biopsy and clinical data use consent before the renal biopsy was performed, which included the agreement on the collection of their clinical data and use for the further studies.

Data disaggregated by year and disease type were saved in comma-separated values format. All data were analyzed and visualized using R software (version 3.6.1). Patient ages (continuous variables) expressed as mean ± standard deviation, and analysis of variance or Kruskal–Wallis tests (after examining the normal distribution or homogeneity of variance in each group of data) was used to calculate significant differences. Disease types and sex (categorical variables) are expressed as frequencies or percentages and were compared using either the χ^2^ test or Fisher’s exact test, as appropriate. Differences between periods were evaluated using the χ^2^ test. Statistical significance was set at *p* < 0.05.

## 3. Results

### 3.1. Study participants

A total of 2906 renal biopsy specimens were reviewed; 45 patients who underwent repeat biopsies, 36 patients who had biopsies containing <10 glomeruli, and 22 patients aged <14 years were excluded. Ultimately, 2803 patients were finally included in the study.

The mean age of patients was 40.99 ± 15.78 (mean ± standard deviation) and it increased over time (34.96 ± 13.66, 40.21 ± 15.97, and 44.57 ± 15.61 in periods 1, 2, and 3, respectively). The male-to-female ratio was 1.08:1 (male, 51.98%). Further, the number of biopsy cases increased from 607 in period 1 to 1231 in period 3, and the percentage of patients aged ≥60 years increased from 4.94% in period 1 to 20.07% in period 3 (Table 1).

**Table 1.** Demographic characteristics of patients who underwent renal biopsy.

	2010–2012 (*n* = 607)	2013–2015 (*n* = 965)	2016–2018 (*n* = 1231)	Total (*n* = 2803)
Sex				
Male, *n* (%)	302 (49.75%)	527 (54.61%)	628 (51.02%)	1457 (51.98%)
Female, *n* (%)	305 (50.25%)	438 (45.39%)	603 (48.98%)	1346 (48.02%)
Age, mean ± SD, years	34.96 ± 13.66	40.21 ± 15.97	44.57 ± 15.61	40.99 ± 15.78
Age category				
14–24 years, *n* (%)	182 (29.98%)	201 (20.83%)	149 (12.10%)	532 (18.98%)
25–44 years, *n* (%)	275 (45.30%)	379 (39.27%)	441 (35.82%)	1095 (39.07%)
45–59 years, *n* (%)	120 (19.77%)	247 (25.60%)	394 (32.01%)	761 (27.15%)
≥60 years, *n* (%)	30 (4.95%)	138 (14.30%)	247 (20.07%)	415 (14.80%)
Disease classification				
PGN	477 (78.58%)	827 (85.70%)	990 (80.42%)	2294 (81.84%)
SGN	125 (20.59%)	121 (12.54%)	185 (15.03%)	431 (15.38%)
TIN	1 (0.16%)	6 (0.62%)	27 (2.19%)	34 (1.21%)
Others	4 (0.66%)	11 (1.14%)	29 (2.36%)	44 (1.57%)

PGN: primary glomerulonephritis; SGN: secondary glomerulonephritis; TIN: tubulointerstitial nephritis.

PGN, SGN, TIN, and other diagnoses accounted for 81.8%, 15.9%, 1.6%, and 1.6% of cases, respectively. The proportions of PGN in different periods were 78.58% (period 1), 85.70% (period 2), and 80.42% (period 3); SGN accounted for 20.59% (period 1), 12.54% (period 2), and 15.38% (period 3); and TIN accounted for 0.16% (period 1), 0.62% (period 2), and 2.19% (period 3). The proportion of PGN in different periods showed a growth trend (*p* < 0.05), while the proportions of SGN declined (*p* < 0.01). The proportions of PGN in different age categories were 78% (≤14 years), 81% (15–44 years), 82% (45–59 years), and 85% (≥60 years), while SGNs accounted for 18.98% (≤14 years), 16.07% (15–44 years), 14.06% (45–59 years), and 11.33% (≥60 years).

### 3.2. Overall changes in renal disease

**Figure 1.**
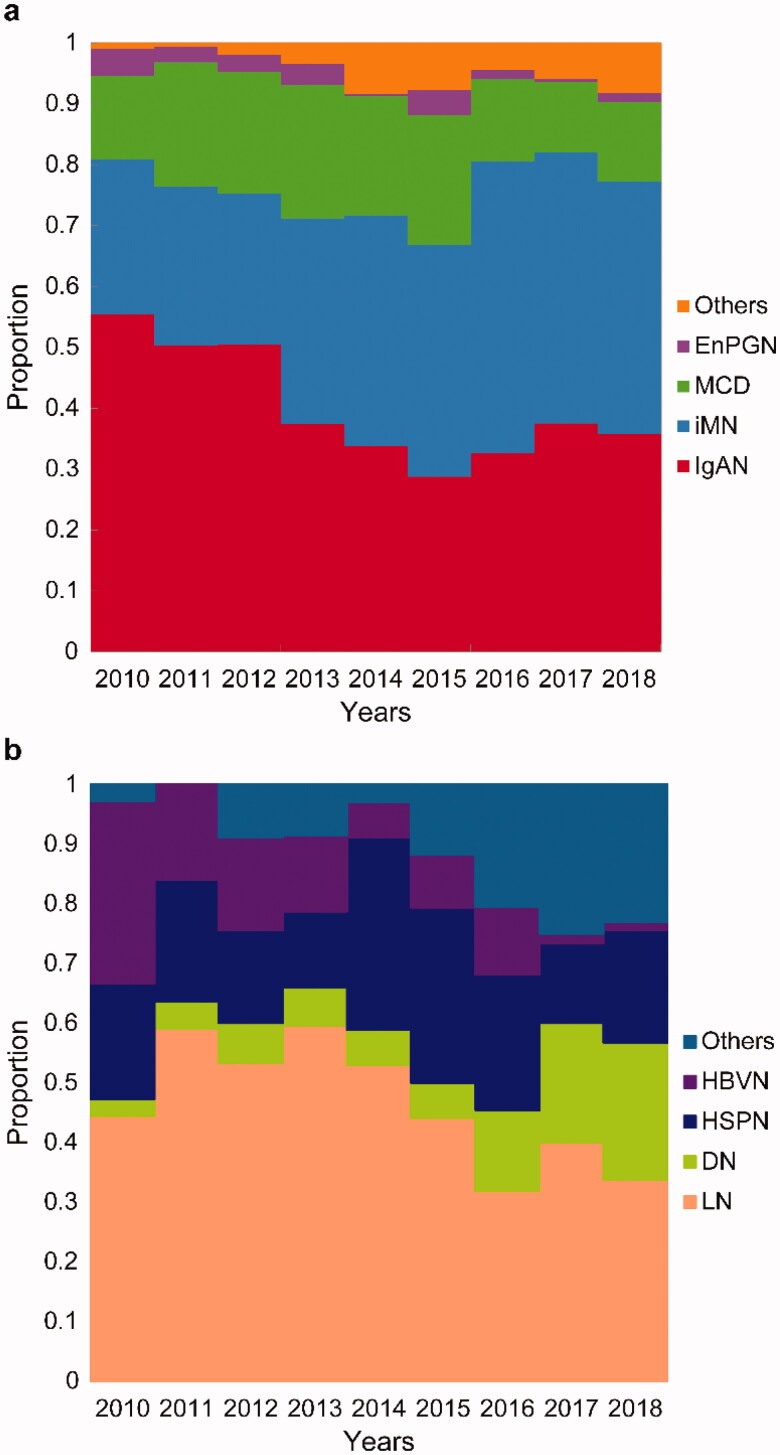
Temporal trends in the frequencies of kidney disease subtypes according to renal biopsy results (2010–2018). (a) Frequencies of PGN subtypes. Proportion of all PGN categories. (b) Frequencies of SGN subtypes. Proportion of all SGN categories. PGN: primary glomerulonephritis; SGN: secondary glomerulonephritis; IgAN: IgA nephropathy; MCD: minimal change disease; iMN: idiopathic membranous nephropathy; EnPGN: endocapillary proliferative glomerulonephritis; LN: lupus nephritis; DN: diabetic nephropathy; HSPN: Henoch–Schönlein purpura nephritis; HBVN: hepatitis B–related nephritis.

**Figure 2.**
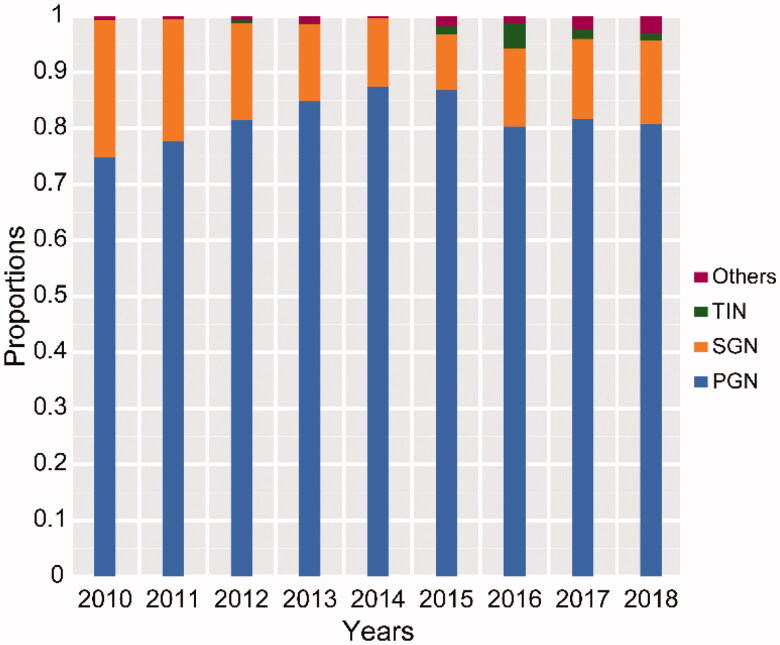
Composition ratio of kidney diseases each year from 2010 to 2018. PGN: primary glomerulonephritis; SGN: secondary glomerulonephritis; TIN: tubulointerstitial nephritis.

IgAN, iMN, and MCD were the main causes of PGN, accounting for 38.06%, 37.49%, and 16.91% of total PGN patients, respectively, from 2010 to 2018. As shown in Online Resource 1, the proportion of iMN significantly increased (25.37% in period 1, 36.52% in period 2, and 44.14% in period 3 [*p* < 0.001]) and nearly doubled from 2010 to 2018. Meanwhile, the frequencies of IgAN, MCD, and EnPGN significantly decreased; the proportion of IgAN was 51.57% in period 1, 33.25% in period 2, and 35.56% in period 3 (*p* < 0.001); the proportion of MCD was 18.66% in period 1, 21.04% in period 2, and 12.63% in period 3 (*p* < 0.001); and the proportion of EnPGN was 3.14% in period 1, 2.78% in period 2, and 1.21% in period 3 (*p* < 0.05) (Online Resource 1, Figure 1(a)). As shown in Online Resource 2, LN, HSPN, DN, and HBVN were the main causes of SGN, accounting for 45%, 19%, 11%, and 10%, respectively. From 2010 to 2018, the proportion of LN decreased significantly (52.8% in period 1, 53.04% in period 2, and 35.39% in period 3; *p* < 0.01) and the proportion of LN in 2018 was nearly half of the highest proportion in 2011 (Figure 1(b)). Further, the proportion of HBVN declined significantly from 30.56% in 2010 to 1.35% in 2018 (*p* < 0.001) and was most greatly decreased in the 14–24 years age category. However, the proportion of DN increased significantly from 4.80% in period 1 to 19.66% in period 3 (four times higher than that in period 1) and increased with increasing age. The proportion of HSPN maintained a steady state in different periods and had the highest incidence in the 14–24 years age category (Online Resource 2, Figure 1(b)). From 2010 to 2018, the PGN/SGN ratio did not change significantly; PGN was still the main subtype of renal biopsy disease at our center. The highest PGN/SGN ratio was observed in 2015 (8.22:1), and the lowest ratio was observed in 2010 (3.05:1) (Figure 2).

### 3.3. Changes in the frequency of renal diseases according to age and sex

**Figure 3.**
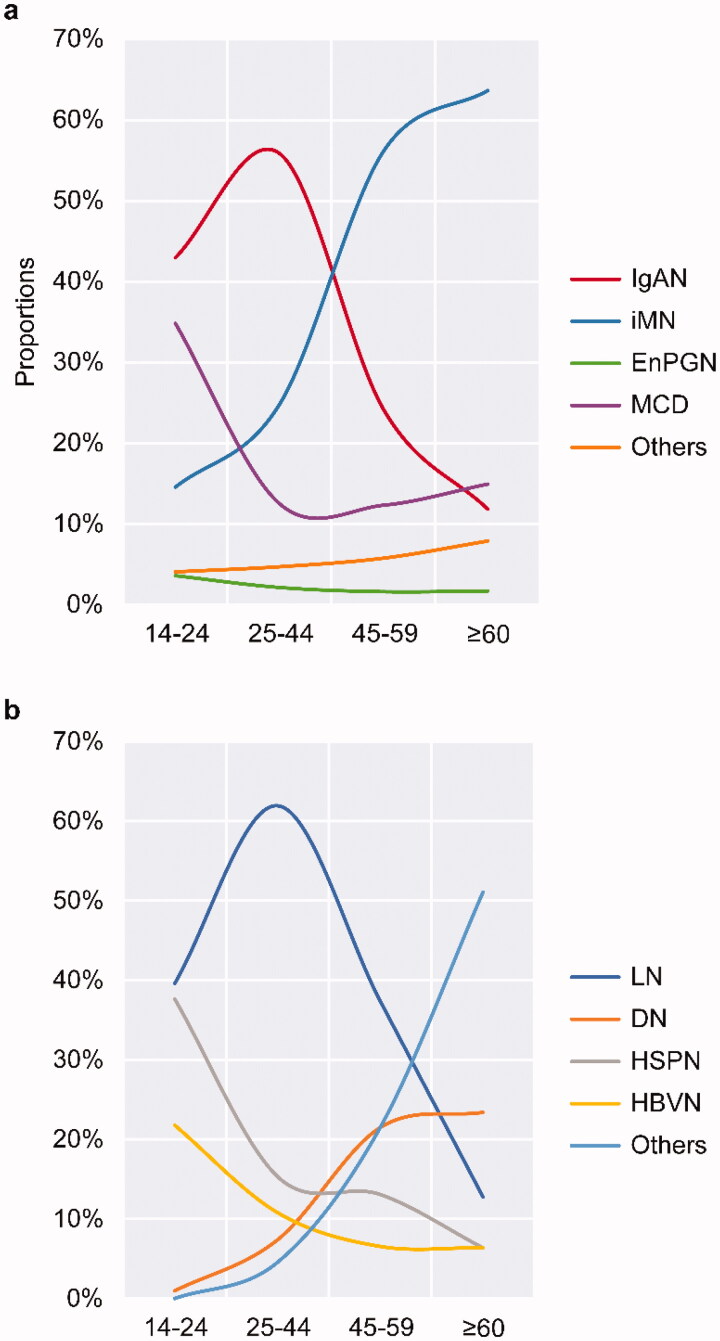
Variations in the tendencies of renal biopsy subtypes according to age. (a) Variation tendency of primary glomerulonephritis subtypes by age. (b) Variation tendency of secondary glomerulonephritis subtypes by age. IgAN: IgA nephropathy; MCD: minimal change disease; iMN: idiopathic membranous nephropathy; EnPGN: endocapillary proliferative glomerulonephritis; LN: lupus nephritis; DN: diabetic nephropathy; HSPN: Henoch–Schönlein purpura nephritis; HBVN: hepatitis B–related nephritis.

**Figure 4.**
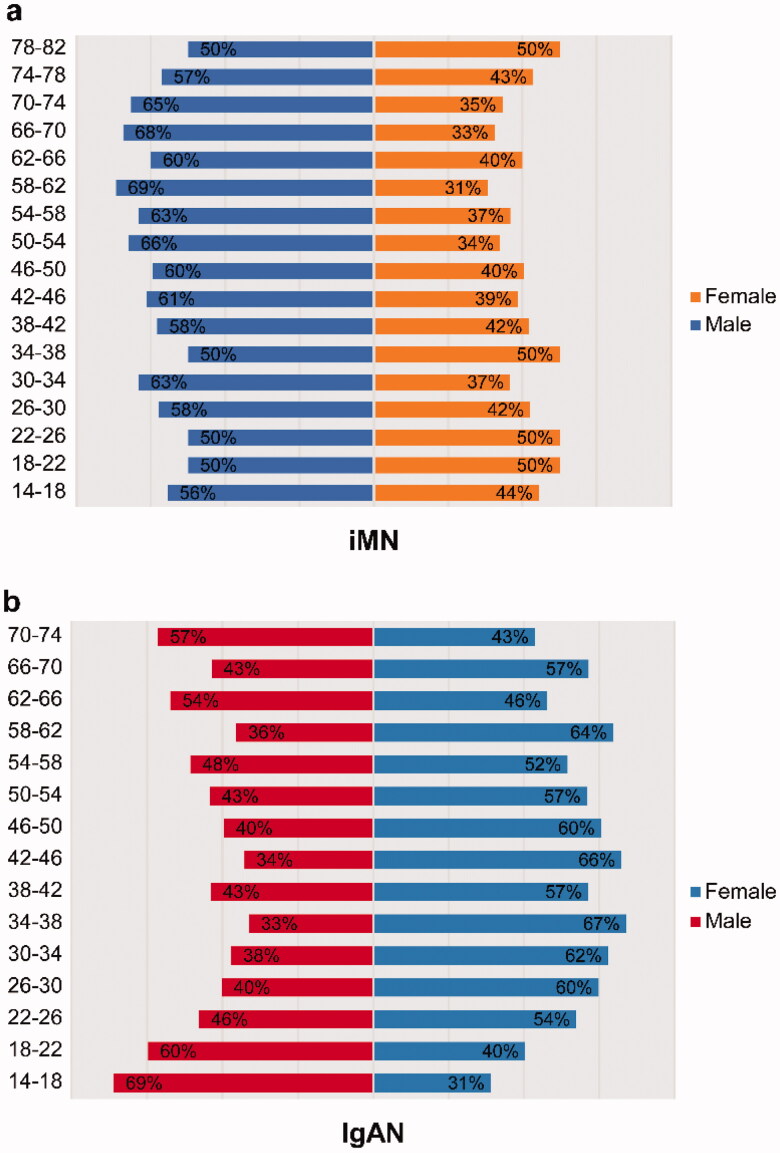
Variations in the sex ratio according to age for iMN (a) and IgAN (b). iMN: idiopathic membranous nephropathy; IgAN: IgA nephropathy.

IgAN, iMN, and MCD were the leading PGN diagnoses at our center. The proportions of these main subtypes changed significantly according to age. Specifically, IgAN mostly occurred in young patients with a peak in the 25–44 years age category (55.87%); the incidence then dropped sharply to 11.83% in the ≥60 years age category, which means that nearly 60% of young PGN patients were diagnosed with IgAN. The highest frequency of MCD was observed in the 14–24 years age category, which accounted for 34.84% of the cases, and then decreased with increasing age, despite the slightly elevated frequency in the ≥60 years age category. The constituent advantage of iMN increased gradually and reached in the ≥60 years age category, indicating that 63.66% of the patients aged ≥60 years had iMN (Figure 3(a)). On the analysis of the sex ratio according to age, we found that men were more vulnerable to the effects of iMN in all age categories (Figure 4(a)). However, IgAN was more common among women in the 34–38 years age category (Figure 4(b)).

Overall, LN, HSPN, DN, and HBVN were the most common SGN diagnoses. Statistical analysis revealed significant differences in the frequency of each SGN in different age categories. The frequency of LN reached a peak (61.93%) in the 25–44 years age category and then dropped sharply to 1.45% in patients aged ≥60 years, indicating that >60% of young SGN patients had LN. The frequency of both HSPN and HBVN decreased with age, peaking in patients aged 14–24 years (37.62% for HSPN and 21.78% for HBV). However, the frequency of DN increased with age and reached a plateau after age 45 years. The frequency of other subtypes of SGN increased dramatically with age and reached a peak of 51.06% at age ≥60 years; renal amyloidosis accounted for most cases in the ≥60-year age group (Figure 3(b)).

## 4. Discussion

In this study, we analyzed and summarized renal disease characteristics and the changing spectrum using renal biopsy. Results revealed that PGN accounted for 81.84% of patients in whom renal biopsy was performed, indicating that it is still the most common renal disease among renal biopsy patients in Jiangxi province, while the frequency of factors contributing to secondary kidney disease increased with each passing year. There is a clear trend of increased mean age of people receiving renal biopsy; probably most attributable to the improved biopsy technologies due to which advanced age is no longer a contraindication for renal biopsy [11]. Besides, the aging population in China might also be an important reason [12,13]. In PGN, the frequency of iMN has increased considerably from 2010 to 2018 and occurred most frequently in the older group; the proportion of male patients was higher than that of female patients in nearly all age categories. Meanwhile, the frequency of IgAN decreased significantly from 51.57 to 35.56%, with a maximum frequency in the 25–44 years age category. LN was the leading cause of SGN (52.80%); however, the frequency decreased significantly with the increasing frequency of DN. Moreover, the frequency of HBVN decreased dramatically over the past 10 years.

In accordance with other studies in China [14–16], our main finding was the increasing frequency of iMN; the proportion of iMN increased from 25.37% in period 1 to 44.14% in period 3. Thus, instead of IgAN, iMN was found to be the most common PGN in Jiangxi province. Liu et al. reported that the frequency of iMN doubled from 10.41 to 24.10% between 2003 and 2014 at their center, which is also located in southern China [14]. However, the frequency of iMN in northern China ranged from 16.8 to 29.35% between 2003 and 2012 [7]. Although the growth rate in our study was nearly the same as that in other studies in China, the frequency of iMN at our center was much higher. This might be caused by the differences in survey periods between our study and previous studies, which indicate that the frequency of iMN continues to increase after 2014. Furthermore, the selection criteria of cases for conducting renal biopsy differ between centers in different regions, which is another cause of the difference in the frequency of iMN. By contrast, the proportion of iMN in Japan [17], Korea [18], the USA [19], and the UK [20] has decreased. In addition to geographical and genetic factors, studies have revealed that environmental pollution, especially exposure to high levels of particulate matter 2.5, is closely related to the increased risk of iMN in China [21].

In our study, the frequency of iMN increased with time in each age category, although the remarkable rising trend was in the ≥60 years age category, which is different from the findings of Hou JH et al. Hou JH et al. reported that the frequency of iMN in the 14–24 years age category increased from 4.91 to 10.56%, which was the highest increase among all age categories. The authors speculated that this was because younger patients are more vulnerable to air pollution [14]. Our center is located in southeast China where there are fewer industries and less air pollution. Therefore, the increase in iMN in this area is more related to population aging, as the highest increasing proportion of iMN in our study was found in older patients. The male-to-female ratio of iMN in our study was 1.6, and the proportion of male patients with iMN was higher in nearly all age categories. This is consistent with the findings of Zhu et al., who indicated a male-to-female ratio of 1.47 in a total of 231 iMN patients [22]. Further, several studies have stated that the male sex is a predictor of progression to end-stage renal disease in iMN [23,24].

Consistent with previous studies of Chinese subpopulations, the frequency of IgAN significantly declined over time [15,16], although IgAN was still the second most common PGN at our center. However, the frequency of IgAN in other countries, such as the USA [8] and Japan [17], remains stable. Further, the frequency of MCD declined during the past 10 years at our center, which may be due to changes in diagnostic and treatment strategies. According to the KDIGO guideline for glomerulonephritis published in 2012, young patients especially those aged <18 years who are diagnosed with nephrotic syndrome are recommended to use corticosteroid as an initial treatment before renal biopsy [25]. Most patients can reach clinical remission after treatment. Renal biopsy is conducted only in cases of frequent relapse or steroid-dependent/resistant young nephrotic syndrome patients [26].

In SGN cases, renal damage secondary to diabetes tended to occur more frequently, while the incidence of HBVN and LN decreased significantly. Over the past few decades, there has been a surge in patients with diabetes in China, leading to an increased incidence of DN [26], which will likely continue. In the southeastern USA, the number of renal biopsies diagnosed as DN has increased over the past few decades [8]. Usually, there is either no proteinuria or a small amount of proteinuria in early diabetes. In addition, many patients who were diagnosed with diabetes did not undergo renal biopsy unless they experienced nephropathy symptoms. Therefore, the incidence of DN might be underestimated in clinical settings. Even still, the proportion of patients with DN increased over time in the 14–24 years age category, indicating that DN may be occurring at a younger age more often than previously thought. Over time, the incidence of HBVN has decreased, suggesting that China has made great progress in the prevention and control of HBV, as noted in a previous study [27]. This also shows that the proportion of kidney diseases caused by infectious diseases has decreased. The decreased incidence of LN in this study may also partially result from selection bias as patients who underwent renal biopsy had more severe impaired renal function.

This single-center retrospective study has several limitations. First, as a retrospective study, the criteria for renal biopsy may have changed with advances in diagnostic techniques, which may have caused changes in the spectrum of certain diseases. In addition, since the patients were enrolled from a single center, they may only be representative of the regional population. Notably, this study only reflects the incidence of kidney disease in patients who undergo renal biopsy; thus, these findings may not apply to the general population.

PGN is the main type of kidney disease at our center; IgAN used to be the most common, but has now been replaced by iMN. Because of the aging population in our country, the frequency of iMN increased significantly, while that of IgAN, MCD, and EnPGN decreased significantly from 2010 to 2018. Among SGN, DN increased significantly, whereas LN and HBVN levels decreased significantly from 2010 to 2018. Overall, the spectrum of changes in renal disease as presented in this study may serve as a reference for the patient guidance, disease prevention, and future public health interventions.

## Supplementary Material

Supplemental Material

Supplemental Material

## Data Availability

The data that support the findings of this study are openly available at https://www.jianguoyun.com/p/DVLSTQ8Q9NrWCRiLu4EE.

## Online Supplementary Resources

**Online Supplementary Resource 1** Proportions of patients with primary glomerulonephritis in different age categories.

** Online Supplementary Resource 2** Proportions of patients with secondary glomerulonephritis in different age categories. 
