# Supplementary material for: Changes in the spectrum of kidney diseases: a survey of 2803 patients from 2010 to 2018 at a single center in southeastern China
Source: Ren Fail. 2022 Jun 3;44(1):987–93. doi: 10.1080/0886022X.2022.2083517 (PMC9176642; doi:10.1080/0886022X.2022.2083517)
Supplement: Supplemental Material [file IRNF_A_2083517_SM5812.pdf]

Supplementary data 2. The prevalence of second glomerular diseases in different age subgroups

Subgroup (years)		2010-2012 N (%)	2013-2015 N (%)	2016-2018 N (%)	P-value
14-24	LN	17(34.69%)	11(50.00%)	10(38.46%)	0.472276
	DN	0(0.00%)	0(0.00%)	1(3.85%)	0.264233
	HSPN	16(32.65%)	8(36.36%)	14(53.85%)	0.192408
	HBVN	16(32.65%)	3(13.64%)	1(3.85%)	<0.01
	Others	0(0.00%)	0(0.00%)	0(0.00%)	
25-44	LN	39(75.00%)	37(67.27%)	30(50.00%)	<0.05
	DN	2(3.85%)	1(1.82%)	10(16.67%)	<0.01
	HSPN	4(7.69%)	13(23.64%)	10(16.67%)	0.080844
	HBVN	5(9.62%)	4(7.27%)	4(6.67%)	0.836494
	Others	2(3.85%)	0(0.00%)	6(10.00%)	<0.05
45-59	LN	10(47.62%)	13(50.00%)	17(28.33%)	0.090387
	DN	3(14.29%)	3(11.54%)	17(28.33%)	0.133652
	HSPN	3(14.29%)	4(15.38%)	7(11.67%)	0.882093
	HBVN	3(14.29%)	2(7.69%)	2(3.33%)	0.241172
	Others	2(9.52%)	4(15.38%)	17(28.33%)	0.115726
≥60	LN	0(0.00%)	0(0.00%)	6(18.75%)	0.081476
	DN	1(33.33%)	3(25.00%)	7(21.88%)	0.89982
	HSPN	0(0.00%)	2(16.67%)	1(3.13%)	0.272511
	HBVN	1(33.33%)	2(16.67%)	0(0.00%)	<0.05
	Others	1(33.33%)	5(41.67%)	18(56.25%)	0.561377
