# Supplementary material for: Changes in the spectrum of kidney diseases: a survey of 2803 patients from 2010 to 2018 at a single center in southeastern China
Source: Ren Fail. 2022 Jun 3;44(1):987–93. doi: 10.1080/0886022X.2022.2083517 (PMC9176642; doi:10.1080/0886022X.2022.2083517)
Supplement: Supplemental Material [file IRNF_A_2083517_SM5805.pdf]

Supplementary data 1. The prevalence of primary glomerular diseases in different age subgroups

Subgroup (years)		2010-2012 N (%)	2013-2015 N (%)	2016-2018 N (%)	P-value
14-24	IgAN	62(46.62%)	66(38.37%)	52(45.61%)	0.28212
	MN	20(15.04%)	20(11.63%)	21(18.42%)	0.27535
	MCD	44(33.08%)	73(42.44%)	29(25.44%)	<0.05
	EnPGN	6(4.51%)	6(3.49%)	3(2.63%)	0.72781
	Others	1(0.75%)	7(4.07%)	9(7.89%)	<0.05
25-44	IgAN	147(67.12%)	153(48.11%)	200(55.87%)	<0.001
	MN	44(20.09%)	87(27.36%)	91(25.42%)	0.15011
	MCD	20(9.13%)	52(16.35%)	40(11.17%)	<0.05
	EnPGN	6(2.74%)	8(2.52%)	5(1.40%)	0.46164
	Others	2(0.91%)	18(5.66%)	22(6.15%)	<0.01
45-59	IgAN	29(29.59%)	46(21.60%)	76(24.20%)	0.30998
	MN	49(50.00%)	111(52.11%)	191(60.83%)	0.05765
	MCD	17(17.35%)	31(14.55%)	29(9.24%)	<0.05
	EnPGN	2(2.04%)	6(2.82%)	2(0.64%)	0.13710
	Others	1(1.02%)	19(8.92%)	16(5.10%)	<0.05
≥60	IgAN	8(29.63%)	10(8.06%)	24(11.76%)	<0.01
	MN	8(29.63%)	84(67.74%)	134(65.69%)	<0.001
	MCD	8(29.63%)	18(14.52%)	27(13.24%)	0.07920
	EnPGN	1(3.70%)	3(2.42%)	2(0.98%)	0.43298
	Others	2(7.41%)	9(7.26%)	17(8.33%)	0.93613
